# Modeling of lung-liver interaction during infection in a human fluidic organ-on-a-chip

**DOI:** 10.1038/s41598-025-22682-z

**Published:** 2025-10-09

**Authors:** Susanne Reinhold, Christian Herr, Yiwen Yao, Mehdi Pourrostami, Felix Ritzmann, Thorsten Lehr, Dominik Selzer, Yvonne Kohl, Daniela Yildiz, Hortense Slevogt, Christoph Beisswenger, Robert Bals

**Affiliations:** 1 Department of Internal Medicine V – Pulmonology, Allergology and Critical Care Medicine, Saarland University, Kirrberger Strasse 1, 66421 Homburg, Saar Germany; 2 Helmholtz-Institute for Pharmaceutical Research Saarland – HIPS, Molecular Therapies for Lung Disease, 66123 Saarbrücken, Germany; 3 Clinical Pharmacy, Saarland University, 66123 Saarbrücken, Germany; 4 Fraunhofer Institute for Biomedical Engineering IBMT, 66280 Sulzbach, Germany; 5 Experimental and Clinical Pharmacology and Toxicology, Center for Molecular Signaling (PZMS), Saarland University, 66421 Homburg, Germany; 6 Respiratory Infection Dynamics, Helmholtz Centre for Infection Research - HZI Braunschweig, 38124 Braunschweig, Germany; 7 Department of Respiratory Medicine and Infectious Diseases, Hannover Medical School, German Center for Lung Research (DZL), BREATH, 30625 Hannover, Germany

**Keywords:** Biological techniques, Biomarkers, Molecular medicine

## Abstract

Respiratory infections, including pneumonia and COVID-19, are major causes of global mortality and morbidity. Recent advancements in organ-on-a-chip (OOC) technologies have paved the way for human-based disease models, offering new tools for studying disease mechanisms and accelerating drug development. The aim of this study was to establish a lung-liver fluidic system to study the interaction of both organ modules during infection. A two organ (lung-liver) fluidic system was established using primary human bronchial (HBECs) or alveolar type epithelial cells (AT) for the lung module and Huh-7 cells for the liver module. Inactivated non-typeable *Haemophilus influenzae* (NTHi) and *Pseudomonas aeruginosa* PAO1 (PAO1) were applied to the lung module*.* Secreted mediators were screened by dot-blot analysis and quantified. The impact of bacteria-exposed epithelial cells on the liver cell transcriptome was analyzed via mRNA sequencing. Lung and liver cells established stable cultures in a circulatory fluidic system. Activation of HBECs or ATCs with NTHi or PAO1 resulted in the secretion of multiple inflammatory mediators into the microfluidic medium including tumor necrosis factor-alpha (TNF-α), monocyte chemotactic protein-1 (MCP-1) and macrophage inflammatory protein-1-α (MIP-1α). Addition of lung cells and application of bacterial onto the HBEC module led to significant transcriptomic alterations in the liver cell module. Gene ontology enrichment analysis showed the induction of various pathways involved in host defense, metabolism, repair, and acute phase response. In conclusion, a two-organ lung-liver fluidic system was established to study the interaction of the organ modules during infection. Mediators like cytokines are released from epithelial culture modules into the fluidic circulation after exposure to bacterial pathogens that significantly modify the gene expression patterns of liver cells.

**Supplementary Information:**

The online version contains supplementary material available at 10.1038/s41598-025-22682-z.

## Introduction

Infections of the respiratory tract such as pneumonia or COVID-19 cause high mortality and morbidity worldwide. Infections cause local organ damage and in addition systemic effects in the whole organism, which contribute to the outcome of the disease. In vitro models have been used to study various aspects of the interaction between microorganisms and lung epithelial cells. Further, the predictive power of animal experiments in drug development has been criticized, and it has been proposed that in several aspects human cell models may better reflect the in vivo situation. This has accelerated the model development within the 3R strategy (reduce, refine, replace) to replace animal experiments. However, models allowing to study the interaction between different organs during infectious diseases are limited.

Organ-on-a-chip (OOC) technologies have been developed in the last years with the aims to establish human-based disease models to study basic disease mechanisms and to provide a tool to speed up drug development^1–4^. One important goal of OOC development is to generate models that help in the development of drugs and evaluate the toxicology of diverse substances such as drugs or environmental substances. Nevertheless, it is difficult to model many aspects of intact organisms in a complex in vitro OOC system^3^.

Various systems have been developed for studying lung or liver cells in OOC systems. Culture of lung cells has been developed in the last decades with several areas of interest. Isolation of stem cells for airway or alveolar cells has been developed and the role of the interaction between epithelial cells and the mesenchymal niche have been highlighted^5,6^. Epithelial cells can be cultured in a variety of 2D or 3D approaches comprising conventional submersed, air liquid interface or organoid cultures with or without co-culture with supportive cells. Lung OOC systems have been developed in a variety of setups including mechanical stretching to model ventilatory movements (“breathing lung-on-a-chip”)^7,8^. A small airway OOC system recapitulated chronic obstructive pulmonary disease (COPD) -like inflammatory processes^9,10^. Liver cells have been used in various setups for OOC systems^11^as spheroid cultures ^12^, a sandwich of primary human hepatocytes on coated coverslips covered by Matrigel^13^, or as 2D-culture on membranes within flow channels^14^. Integrating multiple organ systems within a fluidic platform enables the examination of inter-organ interactions, moving closer to the goal of investigating biological processes and drug effects within organism-like structures. Several studies have explored the use of multi-OOC systems, incorporating both lung and liver modules. These systems employed human airway epithelial cells (AECs) in air liquid interface (ALI) cultures alongside HepaRG™ liver spheroids to investigate how liver cells modulate the toxicity of aflatoxin B1^12^. Additional multi-OOC configurations combined intestinal, liver, skin and kidney cells^15,16^, neurospheres and liver cells^12,16–18^. OOC systems have also been applied to study bacterial or viral infectious events^9,19–22^.

Despite the progress in the field, several obstacles slow down the broad application and expansion of OOC technologies in basic and translational science^23^. To generate fully differentiated tissue equivalents, it is necessary to provide optimal growth conditions, which are difficult to establish in most OOC systems. In multi-OOC, multiple parameters need to be considered: individual culture conditions for the individual organ modules, connecting media, fluidic system, presence or absence of additional cells/tissues such as fibroblasts, immune or endothelial cells. Infection models in OOC multi-OOC systems have been rarely established and require additional adjustments such as the application route and dosing of the microorganisms. For lung-OOC systems investigating the alveolar barrier, the use of alveolar type-1/-2 (AT1/2) cells is still difficult because of the spontaneous differentiation of type-2 into type-1 cells in ALI-culture^24^ and the limited proliferative capacity of the terminally differentiated type-1 cells^25^.

The aim of the present study was the evaluation of a lung—liver multi-OOC system in infection models. The liver has been shown to play a central role in metabolizing bacterial products and molecules that are produced during inflammation and lung disease^26,27^. It is perfused by the blood stream connecting it directly to the diseased tissue and thus may play an important role in the progression of the disease. We aimed to characterize how gene expression in liver cells is regulated by infected lung epithelial cells. For the lung cells we applied differentiated ALI cultures of airway and alveolar human primary cells. Liver cells were cultured as a submersed monolayer of Huh-7 cell line, which has been shown to express a variety of cytochrome P450 enzymes and drug transporters^28,29^. We decided to use a commercially available system, which has been used with lung epithelial cells and hepatocytes in pharmacological and toxicological research^13,30,31^.

## Materials and methods

### Cell culture

The hepatocellular carcinoma cell line Huh-7 (Huh-7D 12, Sigma Aldrich Merck, Germany) was cultured at 37 °C and 5% CO_2_ in DMEM-F12 (1:1) (Gibco, Thermo Fisher Scientific, USA) supplemented with 2.5 mM L-glutamine, 10% FBS (Gibco, Thermo Fisher Scientific, USA), and 1% penicillin–streptomycin (Gibco, Thermo Fisher Scientific, USA). Murine 3T3-J2 fibroblasts (Kerafast, USA) were cultured in DMEM supplemented with 10% FBS (Gibco, Thermo Fisher Scientific, USA), 1% penicillin–streptomycin (Gibco, Thermo Fisher Scientific, USA). For the use as feeder cells the fibroblasts were mitotically inactivated with 4 µg/mL mitomycin C in culture medium.

### Primary cell isolation and culture

The use of tissue and cells from patients has been approved by the ethics committee of the Saarland “Landesärztekammer des Saarlandes” and informed consent has been obtained from all patients. All methods were performed in accordance with the relevant guidelines and regulations. Human bronchial epithelial cells (HBEC) were obtained from brush biopsies of patients subjected to bronchoscopy. Isolated cells were expanded in a feeder cell co-culture as described by^32^. Briefly feeder cells were seeded at a density of 2 × 10^4^ cells/cm^2^ in growth medium. On the next day the medium was changed to complete airway epithelial cell growth medium (Promocell, Germany), supplemented with 10 µM Y-27632 (ROCK inhibitor, Tocris Bioscience, UK) and epithelial cells were added.

Human alveolar type epithelial cells (ATC) were isolated from fresh tumor free lung tissue obtained from surgery after informed consent. The tissue was cut in small pieces and dissociated with a GentleMACS dissociator (Miltenyi, Germany). The cell suspension was digested using 5 mL of a solution containing 2.5 mg/mL collagenase type I (Life Tech, Germany), 0.5 mL dispase (Corning, USA), and 1 mg/mL DNase1 (Roche, Germany) for 30 min at 37 °C. The cells were filtered through a 70 µm cell strainer (Miltenyi, Germany) and the erythrocytes were lysed with red-blood cell lysis buffer (ACK, Gibco). The remaining cells were washed twice with 5–10 mL PBS containing 1% FBS (Gibco; USA), and 1 mM EDTA (Sigma-Aldrich; USA). The cells were filtered through a 40 µm cell strainer (Fisher Scientific, USA) and counted via a hemocytometer. ATC cells were further purified with the mouse monoclonal primary antibody HT2-280 (Terrace; USA) and anti-IgM-magnetic beads in a Miltenyi LS column (Miltenyi; Germany) according to the manufacturer’s protocol. Purified cells were seeded in a cell culture flask with AEpiCM-Alveolar Epithelial Cell Medium (ScienCell, USA) with the Epithelial Growth Supplement EpiGCs (ScienCell, USA) and 2% FBS (Thermofisher Scientific, USA).

### Cell differentiation at the air–liquid interface

HBEC or ATC were differentiated at the air–liquid interface as described^33,34^. Briefly, the cells were seeded at a density of 1.5 × 10^5^ cells/well on type I rat tail collagen (Merck, Germany) coated 24 well transwell inserts (Corning 3470, Corning USA). To generate air liquid interface cultures of HBEC, the apical medium was removed and the basolateral medium was changed to the differentiation medium DMEM/ F-12 (1:1) containing 2.5 mM L-glutamine (Gibco, Thermo Fisher Scientific, USA), 2% Ultroser G (Cytogen, Germany), and 0.2% Primocin (InvivoGen, USA). ATC were differentiated under ALI-conditions in AEpiCM (Alveolar Epithelial Cell Medium, ScienCell, USA) with epithelial growth supplement EpiGCs (ScienCell, USA) and 2% FBS (Gibco, Thermo Fisher Scientific, USA). The differentiation of the epithelial cell layer was verified by the transepithelial electrical resistance (TEER) using a Millicell-ERS2 volt ohm meter (EVOM, Merck). Cultures with a TEER of more than 800 Ω × cm^2^ were considered to be differentiated and used in further experiments.

### Cultivation of bacteria

Heat-inactivated non-typeable *Haemophilus influenzae* (clinical isolates, NTHi) was inoculated into 50 mL brain–heart-infusion broth (Roth, Germany) with 1% supplement B (BD Difco™; BD, Germany) and incubated overnight at 37 °C. *Pseudomonas aeruginosa* PAO1 (ATCC 15692, PAO1) was grown in LB-Medium (Carl Roth, Germany) at 37 °C. The bacterial cell pellet was washed with PBS (Sigma-Aldrich, USA), heat-inactivated at 70 °C for 15 min, cooled on ice and lysed with ultrasound for 45 s. The total protein content was analyzed by biochinonic acid-assay (Pierce™ BCA Protein Assay Kit, Thermo Fisher Scientific, USA) and adjusted to 2.5 mg/mL (NTHi) or 1 mg/ml (PAO1). The heat inactivation was verified by plating the lysates on chocolate agar (NTHi) or LB-Agar (PAO1) and incubation for up to 48 h at 37 °C.

### Cell Stimulations

Huh-7 cells were seeded in a 24-well-plate and stimulated with 10 µg/mL heat inactivated NTHi in 250 µL cell culture medium. Fully differentiated HBEC ALIs (24-well) were stimulated with 5 µg/mL heat inactivated NTHi in 250 µL differentiation medium.

After 18 h, the supernatant was removed and the cells used for RNA-isolation. Huh-7 cells were seeded in 24-well-plates and stimulated with 500 µL of differentiation medium containing 50 ng/mL of the inflammatory cytokines TNFα, IL-6 and IL-1ß (all R&D Systems; USA). After 24 h the supernatant was removed and the cells analyzed for gene expression.

### Setup of the lung-liver fluidic system and stimulation

**Fig. 1:**
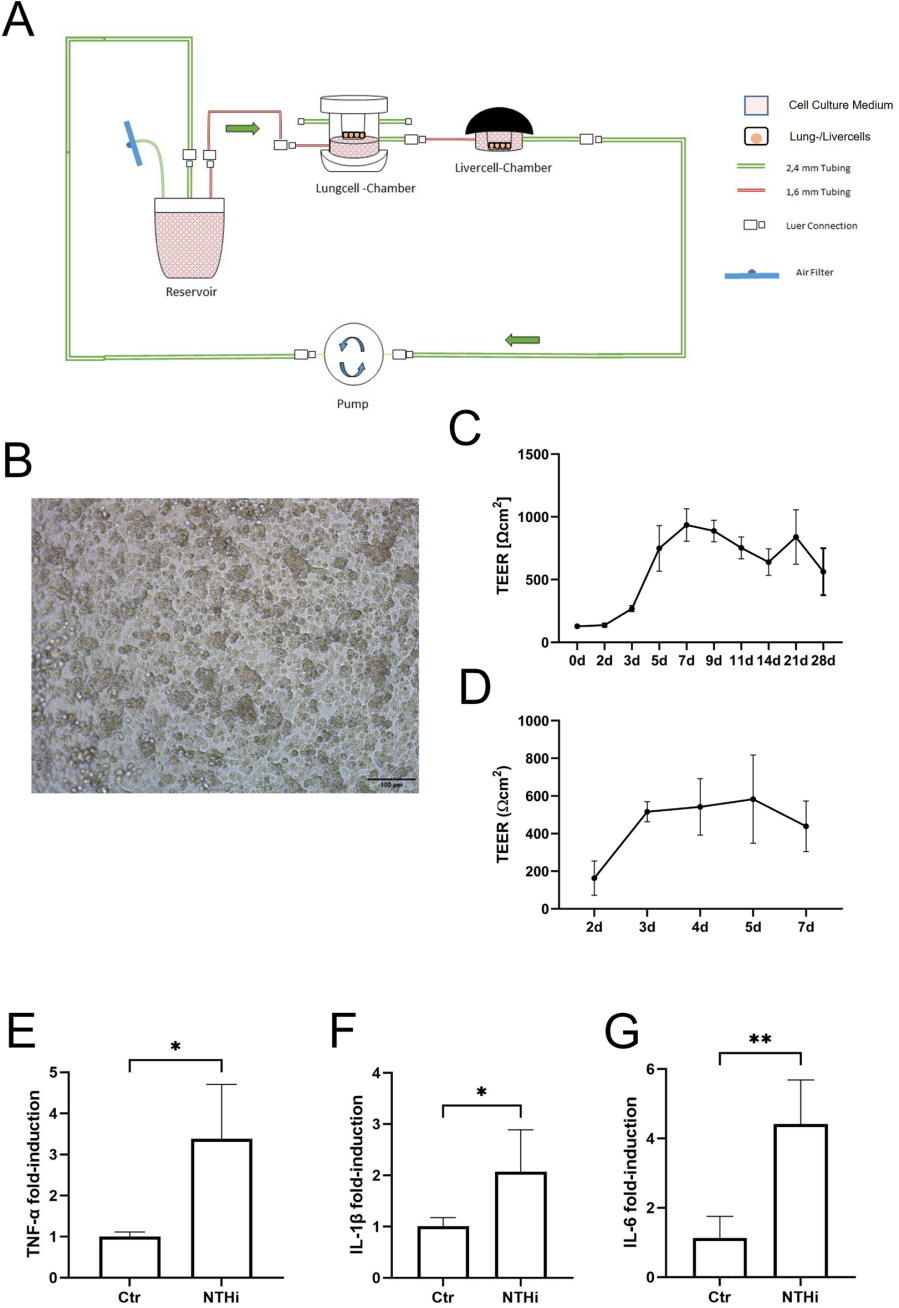
Fluidic lung-liver system setup and the stimulation of HBEC and Huh-7. (**A**) Schematic display of the fluidic system. The tubing connects the lung module, liver module and reservoir supported by a peristaltic Parker PF600 pump. The arrows indicate the direction of the liquid flow, the red part of the silicone tubing has a diameter of 2.4 mm and the green part of 1.6 mm. (**B**) Representative microscopic image of a confluent and differentiated HBEC culture in ALI-conditions. (**C**) Development of TEER of HBEC during differentiation from day 0 to day 21 under ALI-conditions. (**D**) Development of TEER of ATC cultures during differentiation from day 0 to day 7 under ALI-conditions. (**E–G**) Stimulation of HBEC in the fluidic system with NTHi and the gene expression of (**E**) TNF-α, (**F**) IL-1ß, and (**G**) IL-6 24 h after the stimulation. *p < 0.05, **p < 0.01, n = 3, mean values and error bars of the SEM, unpaired Student´s t-test.

A fluidic system from (Quasi Vivo, Kirkstall, UK) was used for the co-culture/stimulation assays and assembled as shown in Fig. 1A. The cell culture chambers consist of silicone and have been used in pharmacological and toxicological research^13,31^. The reservoir was filled with 8 ml of the differentiation media as described above. Differentiated ATC or HBEC on transwells and Huh-7 cells on coverslips were placed into the chambers (QV600 for the HBEC/ATC and QV500 for the Huh-7 cells). The flow rate of the peristaltic pump was adjusted to 500 µL/min and all tubes were flushed with media to remove air. A recent publication showed that flow rates up to 500 µL/min have no impact on surface shear stress in this OOC-model^35^.

Differentiated lung epithelial cells (HBEC or ATC) on transwells were stimulated from the apical side with 20 µL of heat inactivated NTHi or PAOI in differentiation medium (HBEC) or AEpiCM-Alveolar Epithelial Cell Medium (ATC) at the indicated concentrations and the system run for 24 h or 48 h. The medium in the reservoirs was collected and the secretome of the lung cells analyzed by a cytokine-array and a Luminex-Assay. Three independent fluidic circuits were run in parallel at 37 °C and 5% CO_2_ for 24 h or 48 h.

### Characterization of the epithelial secretome by dot-blot- and LUMINEX-analysis

To preselect potential cytokines of interest, the expression profile of inflammatory mediators in the media was analyzed by the Proteome Profiler Human XL-Cytokine Array Kit (R&D Systems, USA) as indicated in the manufacturer’s protocol. The blots were visualized using chemiluminescent detection. The optical densities of the detected analytes were quantified by the software ImageJ 1,53 k (National Institute of Health, USA). A change in optical density of + /− 100 was considered to be upregulated/downregulated.

The concentration of selected mediators was quantified using magnetic premixed human multi-analyte Luminex Kits (Luminex Discovery Assay—R&D Systems, ProcartaPlex™—Thermo Fisher Scientific, USA) as written in the corresponding manual. The assays were analyzed on a MagPix device (Luminex, USA) using xPonent 4.2 (Merck Millipore, USA) and Milliplex Analyst 5.1.0.0 (Vigene Tech, USA).

### RNA-isolation, RT-qPCR, and transcriptome analysis

RNA was extracted using the NucleoSpin RNA kit (Machery-Nagel, Germany) according to the manufacturers protocol. The RNA concentration and quality was determined with a Nanodrop 8000 spectrophotometer (Thermo Fisher Scientific, USA). RNA was subjected to bulk sequencing (DNBSeq PE150, 20 M reads, BGI Tech Solutions, Hong Kong, China).

For the analysis of the gene expression of the selected cytokines or acute phase proteins (APPs) the cDNA was first synthesized using the RevertAid first Strand cDNA-Synthesis kit (Thermofisher Scientific, USA). In the next step RT-qPCR was performed using the SensiMix SYBR& Fluorescein Mix (Meridian; USA) according to the manufacturer’s protocol. Sequences of the primers used are ***SAA1***: forward 5′-CTG CAG AAG TGA TCA GCG-3′ reverse 5′-ATT GTG TAC CCT CTC CCC-3′; ***TNFA:*** forward 5′-TGC ACT TTG GAG TGA TCG GC-3′ reverse 5′-ACT CGG GGT TCG AGA AGA TG-3′; ***IL-1β***: forward 5′-AAC CTA TCT TCT TCG ACA CAT GGG ATA-3′ reverse 5′-CAA GGC CAC AGG TAT TTT GTC ATT ACT-3′; ***IL-6***: forward 5′-CTC AAT ATT AGA GTC TCA ACG CCC A-3′ reverse 5′-GAG AAG GCA ACT GGA CCG AA-3′; ***CRP:*** forward 5′-GAA CTT TCA GCC GAA TAC ATC TTT T-3′ reverse 5′-CCT TCC TCG ACA TGT CTG TCT-3′***; A1AT***: forward 5′-TCA AGG ACA CCG AGG AAG AG-3′ reverse 5′-AGG TGC TGT AGT TTC CCC TC-3′; ***GAPDH***: forward 5′-GTC TCC TCT GAC TTC AAC AGC G-3′, reverse 5′-ACC ACC CTG TTG CTG TAG CCA A-3′. Fold change of gene expression was calculated using the ct-Method after normalization to GAPDH-expression^36^.

### Bioinformatic analysis

For analysis of the mRNA sequencing results, all data was aligned with STAR (version: 2.7.3a) with default parameters, count tables were generated based on gene ID using featureCounts (version: 2.0.1) with default settings. Alignment and gene counts were generated against the GRCh38 genome assembly. DESeq2 package (version: 1.36.0) in R (version: 4.2.1) was used to find differentially expressed genes, the threshold for significance was an adjusted *P* value < 0.05 and log_2_FoldChange >|1|. Heatmaps were generated in R using the pheatmaps package (version: 1.0.12). Functional enrichment analysis, including gene ontology (GO) and Kyoto Encyclopedia Genes and Genomes (KEGG) pathway analysis^37^, were carried out using the R package clusterProfiler (version: 4.4.4).

### Statistical analysis

The same set of devices was used throughout this work after thorough cleaning and autoclaving. Experiments were conducted in parallel such that all experimental conditions to be compared were met. Data analysis was conducted using GraphPad Prism version 10.1.2 (GraphPad Software, San Diego, California, USA). For comparisons involving more than two groups, one-way ANOVA followed by Tukey’s post-hoc test was utilized. When comparing two treatment groups that met parametric assumptions, a Student’s t-test was employed. In instances where parametric assumptions were not satisfied, the Mann–Whitney U test was applied, with specific usage noted in the figure legends. Results were considered as statistically significant with *p* < 0.05.

## Results

### Establishment and validation of the lung liver chip

A two-organ fluidic system was established using cultures of HBEC or ATC for the lung module and Huh-7 cells for the liver module comprising a chamber for conventional transwell-inserts for air–liquid-interface (ALI) cultures and a chamber for cover-slips suited for submerged cell culture. The chambers were connected via silicone tubing to a peristaltic pump and a media-reservoir in a closed loop (Fig. 1A). Conventional microscopy showed that the HBEC cells in the ALI-chamber were grown as a closed epithelial layer (Fig. 1B). We measured the transepithelial electrical resistance (TEER) as an indicator for epithelial integrity and differentiation. Figure 1C and D show the typical development of the TEER for HBEC (Fig. 1C) or ATC (Fig. 1D) cultures, respectively. Cell cultures were used for the experiments after the TEER reached 800 Ω × cm^2^.

### Microbial stimulation of lung cells increases gene expression of inflammatory cytokines

To show the responsiveness of HBEC cultured in ALI for microbial stimulations, we chose to use the clinically relevant bacterial species NTHi. Bacteria were heat-inactivated to avoid overgrowth of the cultures and to allow for long-term analysis. Inactivated bacteria were applied to the apical surface of the lung epithelial cultures and the fluidic system was run for 24 h or 48 h.

RT-qPCR was used to analyze the induction of established host defense and inflammation related genes after the stimulation with NTHi. Compared to the unstimulated control group, the stimulation with 5 µg/mL NTHi caused a significant increase in the expression of TNF-α (3.39-fold, *p* = 0.0113) (Fig. 1E), IL-1ß (2.07-fold, *p* = 0.0451) (Fig. 1F), and IL-6 (4.41-fold, *p* = 0.0036) (Fig. 1G) in HBEC indicating the cells are responsive to microbial stimulation.

### Stimulation with inflammatory cytokines but not direct bacterial stimulation activates Huh-7 liver cells

**Fig. 2:**
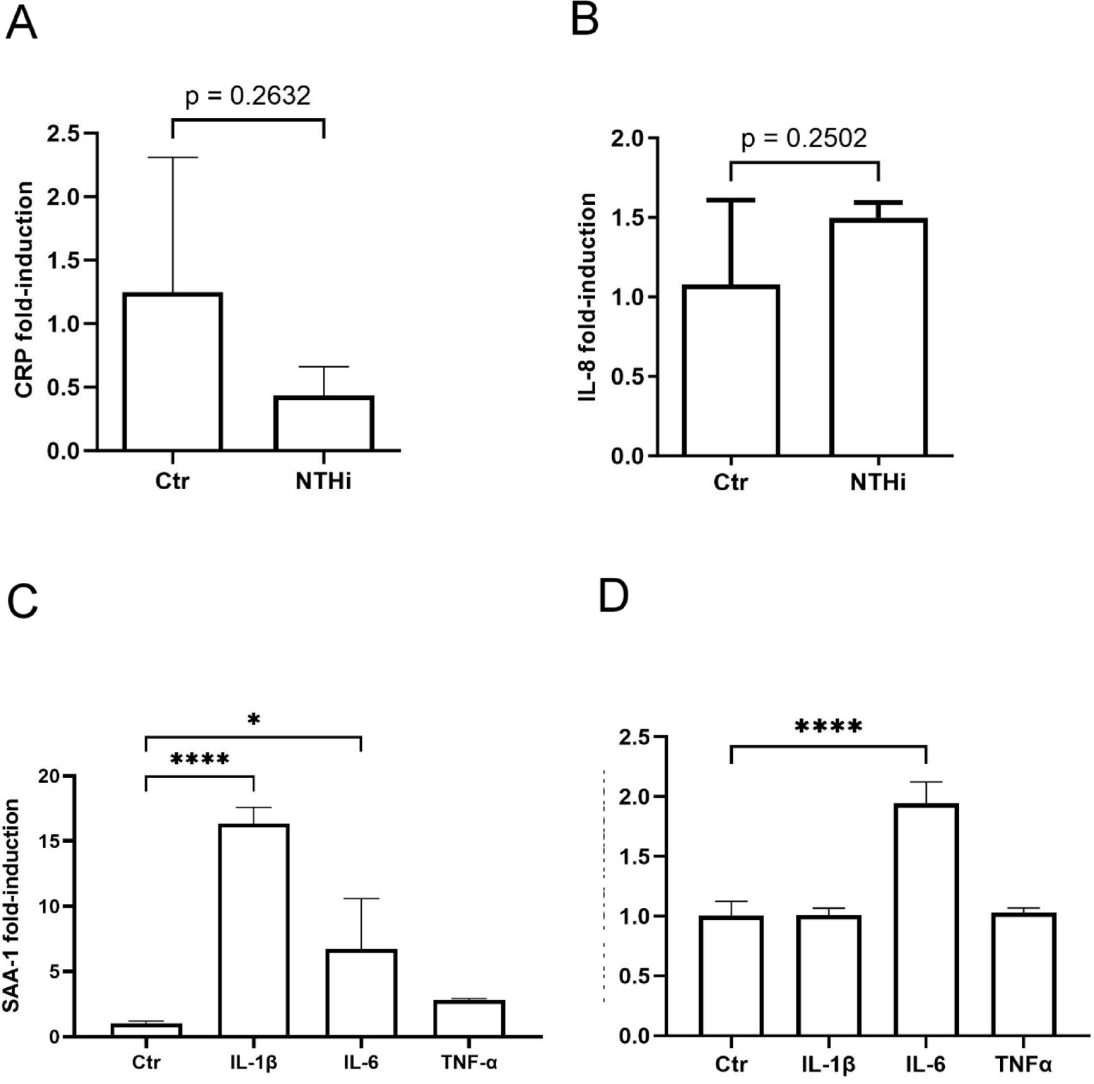
Gene expression of Huh-7 after the stimulation with NTHi and inflammatory cytokines. The gene expression of the acute phase protein CRP (**A**) and the chemokine IL-8 (**B**) 24 h after the stimulation of Huh-7 cells in the fluidic system with NTHi. The gene expression of the acute phase protein SAA-1 (**C**) and α1-antitrypsin (A1AT) (**D**) 24 h after the stimulation of Huh-7 cells in the fluidic system with 50 ng/ml of IL-1β, IL-6, or TNF-α. *p < 0.05; ****p < 0.0001, n = 3. Unpaired Student´s t-test (**A**, **B**) and on way ANOVA with Tukey post hoc test (**C**, **D**).

To evaluate if microbial patterns could directly stimulate liver cells, we tested whether heat-inactivated bacteria could activate Huh-7 cells. Here we also used heat inactivated NTHi only and found that 10 µg/ml NTHi had no detectable effect on the gene expression of CRP (C-reactive protein), a typical acute phase protein of the liver (*p* = 0.2632) (Fig. 2A) or IL-8 (*p* = 0.2502) (Fig. 2B). Only the direct stimulation of Huh-7 cells with the cytokines TNFα, IL-1ß or IL-6 (each 50 ng/mL) resulted in a significant increase in the expression of acute-phase-proteins α-1-antitrypsin (A1AT) and Serum-Amyloid-A1 (SAA-1) (Fig. 2C and D). The expression of SAA-1 was significantly increased after the application of IL-1ß (16.33-fold, *p* =  < 0.0001) and IL-6 (6.7-fold, p = 0.03) (Fig. 2C), whereas the expression of A1AT showed a significant increase only after the stimulation with IL-6 (1.9-fold, *p* = < 0.0001) (Fig. 2D), but not with TNFα or IL-1ß.

### Microbial stimulation of the lung module results in the release of multiple inflammatory mediators

**Fig. 3:**
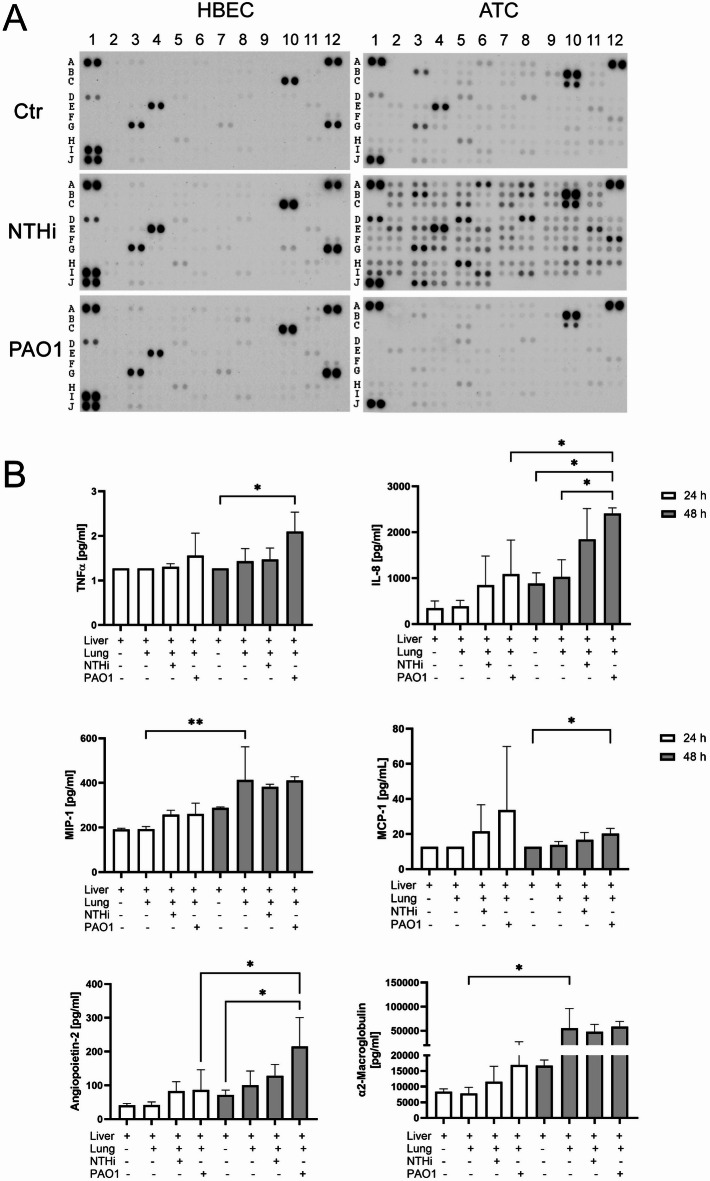
Stimulation of HBEC and ATC with NTHi and PAO1 in the fluidic system connected to Huh-7 liver cells. (**A**) Representative dot-blots of released mediators 24 h after the stimulation of HBEC and ATC with NTHi or PAO1 in the fluidic system connected with Huh-7 liver cells. The corresponding analytes are listed in Tables 1 and 2. (**B**) Quantitative analysis of the stimulation of HBEC in the fluidic system connected to Huh-7 liver cells 24 h and 48 h after the stimulation with NTHi or PAO1. The mediators were analyzed using multiplex Luminex-assays and quantified. *p < 0.05, **p < 0.01, n = 3, mean values and error bars of the SEM, on way ANOVA with Tukey post hoc test.

We characterized the secretome of lung epithelial cells in the cell culture medium of the fluidic system 24 h and 48 h after bacterial exposure of the apical surface and analyzed the cell culture media by a dot-blot cytokine array. Figure 3A shows representative images of the dot blots from HBEC and ATC 24 h after the incubation with 5 µg/ml NTHi and 2 µg/ml PAO1. The densitometric analysis reveals that HBEC were slightly less responsive to bacterial stimulation than ATC (Table 1). We considered a ΔOD of more than ± 100 to be up- or downregulated and based on this calculated the fraction of upregulated and downregulated markers. The detailed values of all markers are included in Supplementary Tables 1 and 2.

**Table 1:** Densitometric evaluation of up- and downregulated markers of the cytokine array. The values were calculated based on a threshold of ΔOD ± 100 and expressed as the fraction of up-and downregulated markers in percent upregulated/downregulated (% ↑ percent upregulated, % ↓ percent downregulated).

	NTHi 24 h		NTHi 48 h		PAO1 24 h		PAO1 48 h	
	% ↑	% ↓	% ↑	% ↓	% ↑	% ↓	% ↑	% ↓
HBEC	4,8	2,9	9,5	18,0	3,8	11,4	8,6	35,2
ATC	89,5	8,6	14,3	30,5	24,8	19,0	13,3	21,9

A co-culture of differentiated HBEC and Huh-7 liver cells was used to investigate the effects of liver cells on the release of biomarkers after the bacterial stimulation of HBEC. HBEC were differentiated in the ALI culture system and placed in the fluidic system connected with Huh-7 liver cells. The HBEC were stimulated from the apical side with 50 µg/mL of heat inactivated NTHi or PAO1.

The stimulation with PAO1 induced a significant increase in the release of TNF-α and MCP-1 after 48 h (Fig. 3B) as compared to liver cells connected to unstimulated HBEC. The release of IL-8 was significantly increased 48 h after the stimulation with PAO1 compared to liver cells and the combination of unstimulated liver and lung cells. MIP-1α was significantly elevated after 48 h compared to 24 h when comparing the combination of liver and lung cells (Fig. 3B). Of interest, significantly increased liver-specific markers like hepatic Angiopoietin-2 and α2-Macroglobulin were detected after the stimulation of the HBEC with NTHi and PAO1 indicating that the lung cells released mediators that stimulated the liver cells (Fig. 3B). The complete analysis is shown in Table 2.

**Table 2:** Circulating mediators 24 and 48 h after bacterial stimulation compared to the unstimulated control group quantified by Luminex assays. Data were compared to the control using one-way ANOVA with Tukey post hoc test, n = 3. Significant values are in [bold].

Analyt [pg/ml]	24 h				48 h			
	Li	LiLu	LiLu_NTHi	LiLu_PAO1	Li	LiLu	LiLu_NTHi	LiLu_PAO1
α2-macro-globulin	8375,33 ± 896,6	7791 ± 1978,87	11,552,67 ± 4959,85	16,878 ± 10,242	16,659,67 ± 1848,13	**55,247,67 ± 33,501,62** **p = 0,0447**	47,711 ± 15,555,45	58,666,33 ± 10,788,39
CRP	19,96 ± 0	19,96 ± 0	19,96 ± 0	19,96 ± 0	19,96 ± 0	19,96 ± 0	19,96 ± 0	19,96 ± 0
MCP-1	12,72 ± 0	12,72 ± 0	21,5 ± 15,21	33,66 ± 36,26	12,72 ± 0	13,83 ± 1,57	16,79 ± 4,09	**20,24 ± 3,02** **p = 0,0385**
MIP-1α	191,52 ± 5,3	193,01 ± 11,82	257,93 ± 19,37	260,7 ± 48,4	287,64 ± 4,3	**413,03 ± 121,61** **p = 0,004**	382,2 ± 11,49	410,82 ± 17,05
IP-10	3,33 ± 0,48	3,21 ± 1,05	4,47 ± 1,09	6,32 ± 3,81	12,03 ± 3,87	60,5 ± 66,99	24,7 ± 7,6	26,06 ± 5,46
IL-1β	3,2 ± 0	3,2 ± 0	3,2 ± 0	3,2 ± 0	3,2 ± 0	3,2 ± 0	3,2 ± 0	3,2 ± 0
IL-12/IL-23 p40	181,23 ± 34,35	192,15 ± 52,92	276,85 ± 37,28	355,13 ± 165,55	677,22 ± 153,58	2107,26 ± 1989,05	1156,01 ± 148,61	1191,33 ± 180,48
IL-1ra	9,18 ± 1,47	110,39 ± 94,15	92,88 ± 61,14	129,89 ± 49,72	26,03 ± 8,71	276,18 ± 55,23	265,98 ± 185,79	207,76 ± 165,07
IL-6	0,87 ± 0	0,87 ± 0	2,13 ± 2,18	1,8 ± 1,62	0,87 ± 0	0,9 ± 0,04	1,76 ± 1,54	2,11 ± 1,95
TNF-α	1,27 ± 0	1,27 ± 0	1,31 ± 0,07	1,56 ± 0,5	1,27 ± 0	1,43 ± 0,23	1,47 ± 0,26	**2,1 ± 0,44** **p = 0,0308**
BDNF	0,88 ± 0	0,88 ± 0	0,88 ± 0	0,88 ± 0	0,88 ± 0	0,88 ± 0	0,88 ± 0	0,88 ± 0
CD31	148,88 ± 0	148,88 ± 0	148,88 ± 0	148,88 ± 0	148,88 ± 0	148,88 ± 0	148,88 ± 0	148,88 ± 0
Eotaxin	0,18 ± 0	0,18 ± 0	0,18 ± 0	0,18 ± 0	0,18 ± 0	0,18 ± 0	0,18 ± 0	0,18 ± 0
IFNγ	3,2 ± 0	3,2 ± 0	3,2 ± 0	3,2 ± 0	3,2 ± 0	3,2 ± 0	3,2 ± 0	3,2 ± 0
Myoglobin	249,91 ± 0	249,91 ± 0	249,91 ± 0	249,91 ± 0	249,91 ± 0	249,91 ± 0	249,91 ± 0	249,91 ± 0
NTpro-BNP	1,67 ± 0	1,67 ± 0	1,67 ± 0	1,67 ± 0	1,67 ± 0	1,67 ± 0	1,67 ± 0	1,86 ± 0,33
PAI-1	447,45 ± 117,15	1547,83 ± 956,51	1335,81 ± 827,52	654,9 ± 375,21	900,16 ± 244,22	3623,38 ± 3453,55	3090 ± 352,49	2186,21 ± 2129,8
S100A8/A9	20,38 ± 0	248,43 ± 388,81	59,01 ± 66,91	47,35 ± 16,95	79,65 ± 75,1	328,88 ± 315,32	340,4 ± 276,05	5321,02 ± 9178,98
SCF	0,24 ± 0	0,24 ± 0	0,24 ± 0	0,24 ± 0	0,24 ± 0	0,24 ± 0	0,24 ± 0	0,37 ± 0,23
Angio-poietin-2	41,31 ± 5,23	41,35 ± 9,41	83,31 ± 27,42	86,93 ± 59,31	72,22 ± 14,1	100,82 ± 34,07	128,69 ± 33,64	**215,02 ± 85,86** **p = 0,0158**
IL-10	1,65 ± 0	1,65 ± 0	1,65 ± 0	1,65 ± 0	1,65 ± 0	2,1 ± 0,64	2,04 ± 0,33	2,47 ± 1,11
IL-18	5,62 ± 2,43	3,74 ± 0,9	8,42 ± 5,48	8,14 ± 1,93	10,09 ± 1,76	9,81 ± 0,8	12,64 ± 1,96	14,92 ± 5,23
IL-8/ CXCL8	345,35 ± 157,36	385,93 ± 129,5	845,43 ± 634,95	1085,65 ± 746,58	884,07 ± 232,48	1029,2 ± 302,77	1845,33 ± 670,6	**2410 ± 122,05** **p = 0,031**
S100A9	6,11 ± 2,13	14,58 ± 6,89	12,53 ± 5,58	12,83 ± 3,87	15,95 ± 8,7	23,96 ± 10,92	31,99 ± 7,18	3154,02 ± 5423,03
VEGF	90,19 ± 8,92	132,14 ± 26,98	222,62 ± 62,19	154,32 ± 45,14	242,33 ± 32,03	622,08 ± 311,16	492,48 ± 101,46	724,35 ± 526,36

### Microbial stimulation of the lung module drives expression of inflammatory and repair genes in the liver cells

We next investigated whether the release of epithelial mediators into the systemic circulation results in changes of gene expression in liver cells. In the liver-lung-fluidic system the HBECs were stimulated with 50 µg/mL heat-inactivated NTHi or PAO1 as described above. To evaluate the impact of unstimulated lung epithelial cells on the gene expression of the liver cells, an empty epithelial cell module was used in connection with the liver-module as a control. 24 h after the stimulation the transcriptome of the liver cells from the following groups was analyzed:

1. Liver cells (Li) vs. liver/lung cells (LiLu)
2. Liver/lung cells (unstimulated) (LiLu) vs. liver/lung cells stimulated with NTHi (LiLu_NTHi)
3. Liver cells/lung cells (unstimulated) (LiLu) vs. liver/lung cells stimulated with PAO1 (LiLu_PAO1)

**Fig. 4:**
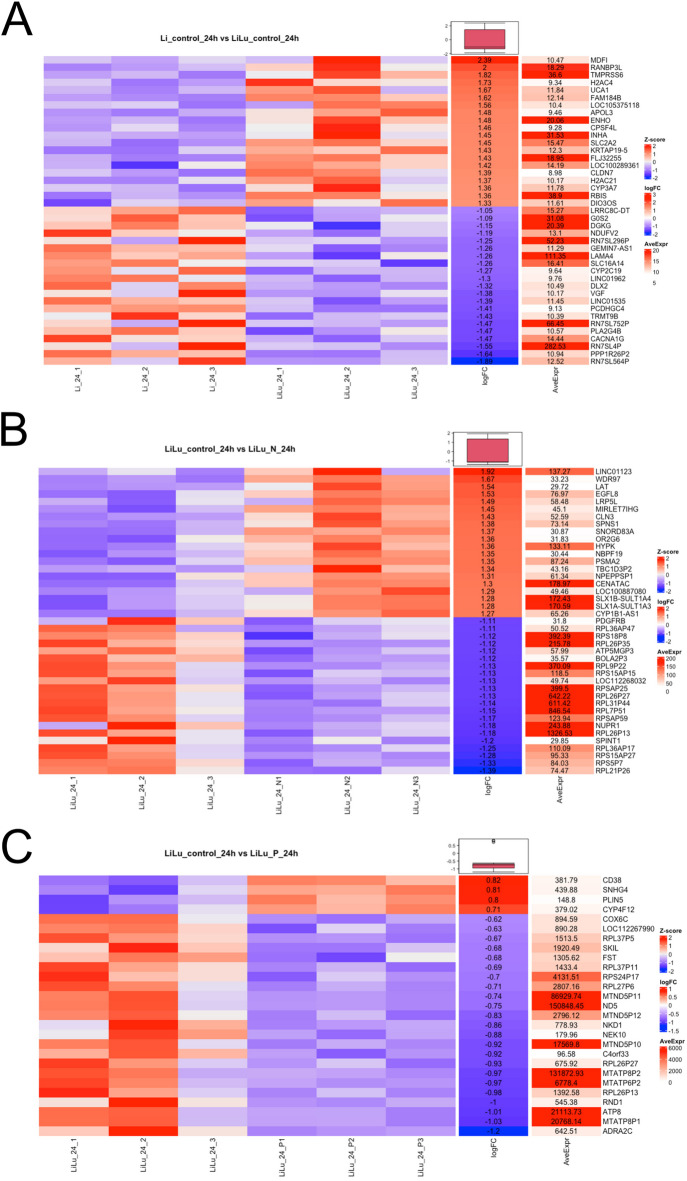
Microbial stimulation of the lung module results in changes of liver cell transcriptome. (**A–C**) Representative heat maps of significantly differentially expressed genes of Huh-7 in the fluidic system connected to differentiated HBEC stimulated with bacteria after 24 h. (**A**) Changes in gene expression of Huh-7 after the addition of unstimulated HBEC (Li_control_24h vs. LiLu_control_24h), (**B**) the stimulation with NTHi (LiLu_24h vs. LiLu_N_24h), and (**C**) the stimulation with PAO1 (LiLu_24h vs. LiLu_P_24h).

First, we analyzed the impact of lung epithelium on liver cell gene expression (Li vs LiLu) and found significant alterations in gene expression. A heat map shows the segregation of the experimental groups (Fig. 4). In the liver cells metabolism associated proteins like RAN-Binding Protein like-3 (RANBP3L), Cytochrome P450 Protein CYP3A7, and SLC2A2 (GLUT2, Glucose Transporter Type 2) were upregulated after the addition of HBEC. The analysis of signaling pathways (KEGG Pathways) showed that cytokine-cytokine-receptor interaction pathway, NF-kB-signaling, and metabolism related pathways like Steroid hormone, Arginine biosynthesis or Retinol metabolism were upregulated.

The microbial stimulation of the lung module with NTHi or PAO1 for 24 h resulted in the modulation of the hepatocyte transcriptome (LiLu vs LiLu_NTHi; LiLu vs. LiLu_PAO1), Fig. 4A–C display the heatmap segregation after the stimulation with PAO1 or NTHi, respectively. The stimulation of HBEC with either PAO1 or NTHi resulted in the upregulation of genes with anti-apoptotic and pro-proliferation properties (HYPK, SNHG4, Fig. 4B and C), genes involved in genome stability or remodeling (SLX1, CENATAC, Fig. 4B) or cell activation (CD38, Fig. 4C). Based on the KEGG-Pathway analysis pathways for metabolism, xenobiotics degradation, and immune system were upregulated in hepatocytes after the stimulation of HBEC with PAO1 or NTHi.

These data show that mediators released from epithelial culture modules into the microfluidic circulation significantly modify the gene expression patterns of liver cells.

## Discussion

The main finding of this study was that lung epithelial and liver cell cultures can be established in an OOC-multi-organ model and that exposure of lung epithelial cells with bacteria activates hepatocytes via secreted mediators. This is the first study that characterized transcriptome regulation in an inter-organ infection model using microbial stimulation.

Different aspects of lung physiology and pathology have been investigated with lung OOC systems including ventilatory movements^7,18^ (“breathing lung-on-a-chip”) and COPD-like inflammatory processes using a small airway OOC system^9^. Toxicity studies have been performed using lung air–liquid-interface cultures with liver spheroids to investigate the detoxification capacity of liver cells for aflatoxin B1^12^. Interaction between lung cells and brain or endothelial cells after viral stimulation has been investigated with OOC systems^8,38^. In the fluidic setup used in the present study, we hypothesize that microbial stimulation of the lung epithelial layer results in the secretion of inflammatory mediators into the circulating fluid. These mediators then stimulate liver cells resulting in the acute-phase transcriptomes.

Application of NTHi or PAO1 to the lung epithelial cells resulted in the secretion of multiple mediators into the fluidic medium as determined by dot-blot screening and subsequent quantification. The released mediators comprised MCP-1, MIP-1α and IL-8 belonging to typical epithelial mediators. Most interestingly, proteins of hepatic origin like α2-macroglobulin and hepatic angiopoietin-2 were detected after the stimulation of HBEC. We applied airway epithelial cells (HBEC) and alveolar cells (ATC) as both cell types are involved in host defense and likely react to microbes differentially^39,40^. The patterns of released mediators were significantly different between the two cell types, highlighting the need to include HBEC and ATC into experimentation to obtain a comprehensive view on lung host defense.

The main focus of this work was to study the interaction of the lung and liver cells. The sole combination of liver cells with native epithelial cells (HBECs) already caused significant modification of the liver cell transcriptome. Application of NTHi and PAO1 to the epithelial module caused a significant change of the transcriptome of the liver cells with activation of multiple genes. Of note, the expression patterns differed significantly between the two inactivated bacterial species after stimulation with the same concentration. This indicates the capability of epithelial cells to discriminate between bacterial ligands of different pathogens.

We investigated whether liver cells could be directly stimulated by microbial patterns applied to the apical side of the lung epithelia and potentially translocate into the fluidic medium. Stimulation of hepatocytes with bacterial suspensions did not result in the induction of APPs. It is therefore unlikely that bacterial patterns directly stimulate hepatocytes and changes in the hepatocyte transcriptome are likely induced by epithelial mediators released into the microfluidic medium.

This study has limitations and strength. We did not use additional cell types such as fibroblasts or endothelial cells. It is known that fibroblasts support the differentiation of epithelial cells of the lung or the liver^41^. Endothelial cells are important for the establishment of the vascular barrier^42^. We decided not to include these cell types to avoid additional complexity in the establishment of the culture conditions. The setup of the multi-organ OOC system and the infection protocols comprised multiple parameters such as culture condition, cellular source, fluidic medium, perfusion parameters and others^43^. Although our investigation covered numerous variables, as detailed in the results section, we did not conduct a systematic evaluation of all possible parameters due to the exceeding large volume of experiments such an approach would require. Instead, our focus was on identifying and establishing key variables critical for cell culture, media composition, and the dosing for bacterial stimulation. We applied heat-inactivated bacteria to better control stimulation conditions and to avoid bacterial overgrowth. In general, the complexity of such multi-organ OOC systems challenges the comparability of various published setups. In the present study we used primary lung epithelial cells to avoid biases caused by cell lines. As the focus of this work was on the role of lung epithelial cells, we used human airway and alveolar cells. The use of primary cells allowed us to use OOC systems to study patient specific properties such as genetic or epigenetic composition of the cells but also induces variability in parameters like TEER due to the heterogeneity of the cell population. Healthy primary hepatocytes are the gold standard for studies of drug metabolism and toxicity but have a very limited availability and show high variability in gene expression between donors^44,45^. The expression of drug metabolizing enzymes and transporters in Huh-7 has been shown before and comparison to primary hepatocytes from different donors showed the presence of these enzymes in the cell line Huh-7^29,45,46^.

Although abnormal liver functions tests have been observed in patients suffering from community acquired pneumonia^26^, there has been no further investigation on the mechanisms. The lung-liver OOC system established in this work may help to investigate this mechanism. A dose–response and time-course for the bacterial stimulation would help to investigate the interaction of lung and liver cells in more detail. In the current setup we used a concentration of inactivated bacteria that will result in an intermediate inflammatory response of lung epithelial cells. The two time points after stimulation were selected based on our experience with lung epithelial cells to investigate the transcriptome and secretome.

In conclusion, we established a lung-liver OOC system to study the response to infection. HBEC and ATC stimulated with typical respiratory pathogens released multiple mediators into the fluidic medium dependent on the cell type and microbial species resulting in distinct patterns of gene induction in hepatocytes.

## Supplementary Information

Below is the link to the electronic supplementary material.

Supplementary Material 1

Supplementary Material 2

## Data Availability

The mRNA sequences used in this study are accessible at Gene Expression Omnibus (GEO, https://www.ncbi.nlm.nih.gov/geo/) with the accession number GSE293875.
