# Supplementary material for: Modeling of lung-liver interaction during infection in a human fluidic organ-on-a-chip
Source: Sci Rep. 2025 Oct 9;15:35241. doi: 10.1038/s41598-025-22682-z (PMC12511430; doi:10.1038/s41598-025-22682-z)
Supplement: Supplementary file 1 — Supplementary Material 1 [file 41598_2025_22682_MOESM1_ESM.docx]

**Supplementary Table 1: Dot blot analysis of mediator patterns released from HBECs.** Optical density of mediators after HBEC-stimulation with NTHi and PAO1 as compared to the control.

|  |  | | | | | | | | |
| --- | --- | --- | --- | --- | --- | --- | --- | --- | --- |
|  |  | 24h | | | | 48h | | | |
| index |  | ΔOD **NTHi** | ↑ ↓ | ΔOD **PAO1** | ↑ ↓ | ΔOD **NTHi** | ↑ ↓ | ΔOD **PAO1** | ↑ ↓ |
| A2 | Adiponectin | 0.0 |  | 666.7 | ↑ | 0.0 |  | -150.0 | ↓ |
| A3 | Apolipoprotein A-1 | -56.7 | ↓ | -50.7 | ↓ | 0.0 |  | 0.0 |  |
| A4 | Angiogenin | 0.0 |  | -160.0 | ↓ | 300.0 | ↑ | 150.0 | ↑ |
| A5 | Angiopoetin-1 | 0.0 |  | -600.0 | ↓ | 0.0 |  | -37.5 | ↓ |
| A6 | Angiopoetin-2 | -80.0 | ↓ | -68.6 | ↓ | 0.0 |  | -75.0 | ↓ |
| A8 | BDNF | 0.0 |  | -88.9 | ↓ | 0.0 |  | -60.0 | ↓ |
| A10 | CD14 | 0.0 |  | 0.0 |  | -54.5 | ↓ | 0.0 |  |
| A11 | CD30 | -74.7 | ↓ | -97.0 | ↓ | 0.0 |  | -37.5 | ↓ |
| B2 | CD40 Ligand | 0.0 |  | 114.3 | ↑ | 0.0 |  | 0.0 |  |
| B3 | Chitinase 3-like 1 | 0.0 |  | -80.0 | ↓ | 0.0 |  | 0.0 |  |
| B4 | CFD | 0.0 |  | 0.0 |  | -300.0 | ↓ | -128.6 | ↓ |
| B5 | CRP | 0.0 |  | 600.0 | ↑ | 0.0 |  | -900.0 | ↓ |
| B6 | Cripto-1 | 0.0 |  | -200.0 | ↓ | 240.0 | ↑ | 0.0 |  |
| B7 | Cystatin C | 0.0 |  | -333.3 | ↓ | 0.0 |  | -214.3 | ↓ |
| B9 | DPPIV | 0.0 |  | -320.0 | ↓ | 75.0 | ↑ | 0.0 |  |
| B10 | EGF | 0.0 |  | -333.3 | ↓ | 0.0 |  | 0.0 |  |
| B11 | Emmprin | -40.0 | ↓ | -40.0 | ↓ | 0.0 |  | 0.0 |  |
| C2 | ENA-78 | -800.0 | ↓ | -400.0 | ↓ | 0.0 |  | 0.0 |  |
| C3 | Endoglin | 0.0 |  | -133.3 | ↓ | 0.0 |  | 0.0 |  |
| C4 | Fas Ligand | 0.0 |  | 0.0 |  | 37.5 | ↑ | -37.5 | ↓ |
| C5 | FGF basic | 0.0 |  | -114.3 | ↓ | -54.5 | ↓ | -27.3 | ↓ |
| C6 | FGF-7 | 0.0 |  | 0.0 |  | -128.6 | ↓ | -128.6 | ↓ |
| C7 | FGF-19 | 0.0 |  | -333.3 | ↓ | 0.0 |  | 0.0 |  |
| C8 | Flt-3 Ligand | 0.0 |  | 0.0 |  | 150.0 | ↑ | 450.0 | ↑ |
| C9 | G-CSF | 36.4 | ↑ | 0.0 |  | 0.0 |  | 60.0 | ↑ |
| C10 | GDF-15 | 1.0 | ↑ | 0.0 |  | 0.0 |  | 0.0 |  |
| C11 | GM-CSF | -57.1 | ↓ | -85.7 | ↓ | 0.0 |  | 0.0 |  |
| D1 | GROα | 22.0 | ↑ | -27.8 | ↓ | 1549.2 | ↑ | 0.0 |  |
| D2 | Growth Hormone | 0.0 |  | -400.0 | ↓ | 0.0 |  | -150.0 | ↓ |
| D3 | HGF | 0.0 |  | 0.0 |  | 75.0 | ↑ | 0.0 |  |
| D4 | ICAM-1 | 1400.0 | ↑ | 600.0 | ↑ | 0.0 |  | 0.0 |  |
| D5 | IFN-γ | -9.3 | ↓ | -93.0 | ↓ | 0.0 |  | -60.0 | ↓ |
| D6 | IGFBP-2 | 0.0 |  | 0.0 |  | 60.0 | ↑ | 60.0 | ↑ |
| D7 | IGFBP-3 | 0.0 |  | 0.0 |  | -1500.0 | ↓ | 0.0 |  |
| D8 | IL-1α | 0.0 |  | -53.7 | ↓ | 0.0 |  | 600.0 | ↑ |
| D9 | IL-1β | 0.0 |  | 0.0 |  | -37.5 | ↓ | 37.5 | ↑ |
| D10 | IL-1 ra | 0.0 |  | 40.0 | ↑ | 0.0 |  | -60.0 | ↓ |
| D11 | IL-2 | 0.0 |  | -8.3 | ↓ | 0.0 |  | 933.3 | ↑ |
| D12 | IL-3 | 0.0 |  | 0.0 |  | -300.0 | ↓ | 0.0 |  |
| E1 | IL-4 | -80.0 | ↓ | 0.0 |  | 75.0 | ↑ | 0.0 |  |
| E2 | IL-5 | 0.0 |  | -40.0 | ↓ | 0.0 |  | -161.5 | ↓ |
| E3 | IL-6 | -96.0 | ↓ | -56.0 | ↓ | 0.0 |  | 0.0 |  |
| E4 | IL-8 | 1.8 | ↑ | 1.8 | ↑ | 0.0 |  | 0.0 |  |
| E5 | IL-10 | 0.0 |  | 0.0 |  | 0.0 |  | -600.0 | ↓ |
| E6 | IL-11 | 0.0 |  | 0.0 |  | 0.0 |  | 450.0 | ↑ |
| E7 | IL-12 p70 | 0.0 |  | 0.0 |  | -138.5 | ↓ | -207.7 | ↓ |
| E8 | IL-13 | 0.0 |  | 0.0 |  | 27.3 | ↑ | -54.5 | ↓ |
| E9 | IL-15 | 0.0 |  | 0.0 |  | 0.0 |  | -85.7 | ↓ |
| E11 | IL-17A | 0.0 |  | -333.3 | ↓ | 0.0 |  | 0.0 |  |
| E12 | IL-18 Bpa | 0.0 |  | 0.0 |  | 150.0 | ↑ | 0.0 |  |
| F3 | IL-23 | 0.0 |  | 0.0 |  | 0.0 |  | -214.3 | ↓ |
| F4 | IL-24 | 109.1 | ↑ | 0.0 |  | 0.0 |  | 0.0 |  |
| F5 | IL-27 | 0.0 |  | 0.0 |  | 0.0 |  | -900.0 | ↓ |
| F6 | IL-31 | 0.0 |  | 0.0 |  | 0.0 |  | -375.0 | ↓ |
| F7 | IL-32 | 0.0 |  | 0.0 |  | 0.0 |  | -180.0 | ↓ |
| F8 | IL-33 | 0.0 |  | 0.0 |  | 112.5 | ↑ | 37.5 | ↑ |
| F9 | IL-34 | 0.0 |  | 0.0 |  | 54.5 | ↑ | -27.3 | ↓ |
| F10 | IP-10 | 0.0 |  | 0.0 |  | 600.0 | ↑ | 900.0 | ↑ |
| F11 | I-TAC | 0.0 |  | 0.0 |  | 0.0 |  | -75.0 | ↓ |
| F12 | Kallikrein-3 | 0.0 |  | -48.5 | ↓ | 0.0 |  | 0.0 |  |
| G1 | Leptin | 0.0 |  | 0.0 |  | -225.0 | ↓ | 0.0 |  |
| G2 | LIF | 0.0 |  | 0.0 |  | -150.0 | ↓ | -131.3 | ↓ |
| G3 | Lipocalin-2 | 2.1 | ↑ | 3.6 | ↑ | 3.3 | ↑ | 0.0 |  |
| G4 | MCP-1 | -31.6 | ↓ | 0.0 |  | 6600.0 | ↑ | 0.0 |  |
| G5 | MCP-3 | 0.0 |  | 0.0 |  | 0.0 |  | -900.0 | ↓ |
| G6 | M-CSF | 0.0 |  | 0.0 |  | -375.0 | ↓ | -450.0 | ↓ |
| G7 | MIF | -93.3 | ↓ | -40.0 | ↓ | 2976.9 | ↑ | -138.5 | ↓ |
| G8 | MIG | 0.0 |  | 0.0 |  | -150.0 | ↓ | 750.0 | ↑ |
| G9 | MIP-1α / MIP-1β | 5000.0 | ↑ | 0.0 |  | -150.0 | ↓ | -225.0 | ↓ |
| G10 | MIP-3α | 4800.0 | ↑ | 0.0 |  | 2228.6 | ↑ | -171.4 | ↓ |
| G11 | MIP-3β | 22.2 | ↑ | 0.0 |  | 60.0 | ↑ | -150.0 | ↓ |
| G12 | MMP-9 | -3.0 | ↓ | -1.8 | ↓ | 0.5 | ↑ | 0.0 |  |
| H1 | Myeloperoxidase | 30.8 | ↑ | 15.4 | ↑ | 0.0 |  | 0.0 |  |
| H2 | Osteopontin | -57.1 | ↓ | -28.6 | ↓ | -187.5 | ↓ | -131.3 | ↓ |
| H3 | PDGF-AA | 52.2 | ↑ | -87.0 | ↓ | 0.0 |  | -144.0 | ↓ |
| H4 | PDGF-AB/BB | 0.0 |  | 0.0 |  | -171.4 | ↓ | -214.3 | ↓ |
| H5 | Pentraxin-3 | 14.1 | ↑ | -52.5 | ↓ | 0.0 |  | -109.1 | ↓ |
| H6 | PF-4 | 0.0 |  | 0.0 |  | -1200.0 | ↓ | -900.0 | ↓ |
| H7 | RAGE | 0.0 |  | 0.0 |  | -1200.0 | ↓ | -900.0 | ↓ |
| H8 | RANTES | 0.0 |  | 0.0 |  | 0.0 |  | 600.0 | ↑ |
| H9 | RBP-4 | -133.3 | ↓ | 0.0 |  | 0.0 |  | 0.0 |  |
| H10 | Relaxin-2 | -200.0 | ↓ | 0.0 |  | 0.0 |  | -330.0 | ↓ |
| H11 | Resistin | -22.2 | ↓ | -22.2 | ↓ | 0.0 |  | -173.7 | ↓ |
| H12 | SDF-1α | 0.0 |  | -90.9 | ↓ | 0.0 |  | 0.0 |  |
| I1 | Serpin E1 | -0.5 | ↓ | 0.8 | ↑ | 0.0 |  | 0.0 |  |
| I2 | SHBG | -54.5 | ↓ | -66.7 | ↓ | 0.0 |  | -225.0 | ↓ |
| I3 | ST2 | 0.0 |  | -88.9 | ↓ | 0.0 |  | -138.5 | ↓ |
| I4 | TARC | 0.0 |  | 0.0 |  | -225.0 | ↓ | -300.0 | ↓ |
| I5 | TFF3 | 0.0 |  | 0.0 |  | 0.0 |  | 450.0 | ↑ |
| I6 | TfR | -76.2 | ↓ | -66.7 | ↓ | -150.0 | ↓ | 0.0 |  |
| I7 | TGF-α | 400.0 | ↑ | 0.0 |  | 0.0 |  | 0.0 |  |
| I8 | Thrombospondin-1 | 0.0 |  | -83.0 | ↓ | -210.0 | ↓ | 0.0 |  |
| I10 | uPAR | 0.0 |  | 0.0 |  | 0.0 |  | -168.8 | ↓ |
| I11 | VEGF | -76.9 | ↓ | 0.0 |  | 0.0 |  | -214.3 | ↓ |
| J3 | Vitamin D BPp | -55.9 | ↓ | -79.6 | ↓ | 0.0 |  | -106.5 | ↓ |
| J4 | CD-31 | 0.0 |  | 0.0 |  | -106.5 | ↓ | -116.1 | ↓ |
| J5 | TIM-3 | 0.0 |  | 0.0 |  | -207.7 | ↓ | -184.6 | ↓ |
| J6 | VCAM-1 | 0.0 |  | 0.0 |  | 0.0 |  | -171.4 | ↓ |
