# Supplementary material for: Modeling of lung-liver interaction during infection in a human fluidic organ-on-a-chip
Source: Sci Rep. 2025 Oct 9;15:35241. doi: 10.1038/s41598-025-22682-z (PMC12511430; doi:10.1038/s41598-025-22682-z)
Supplement: Supplementary file 2 — Supplementary Material 2 [file 41598_2025_22682_MOESM2_ESM.docx]

**Supplementary Table 2: Dot blot analysis of mediator patterns released from ATCs.** Optical density of mediators after stimulation of ATC with NTHi and PAO1 as compared to the control.

|  |  |  |  |  |  |  |  |  |  |
| --- | --- | --- | --- | --- | --- | --- | --- | --- | --- |
|  |  | 24h | | | | 48h | | | |
|  |  | ΔOD **NTHI** | ↑ ↓ | ΔOD **PAO1** | ↑ ↓ | ΔOD **NTHI** | ↑ ↓ | ΔOD **PAO1** | ↑ ↓ |
| A2 | Adiponectin | 691.4 | ↑ | -61.7 | ↓ | 280.0 | ↑ | 680.0 | ↑ |
| A3 | Apolipoprotein A-1 | 1754.9 | ↑ | -9.8 | ↓ | -60.9 | ↓ | -43.5 | ↓ |
| A4 | Angiogenin | 1772.7 | ↑ | -90.9 | ↓ | 800.0 | ↑ | 200.0 | ↑ |
| A5 | Angiopoetin-1 | 3281.3 | ↑ | 375.0 | ↑ | -40.0 | ↓ | -80.0 | ↓ |
| A6 | Angiopoetin-2 | 960.2 | ↑ | 51.1 | ↑ | -25.6 | ↓ | -87.2 | ↓ |
| A7 | BAFF | 2346.2 | ↑ | 134.6 | ↑ | -90.9 | ↓ | -72.7 | ↓ |
| A8 | BDNF | 2065.6 | ↑ | 204.9 | ↑ | -177.8 | ↓ | -88.9 | ↓ |
| A9 | C5/C5a | -5750.0 | ↓ | -500.0 | ↓ | -266.7 | ↓ | 266.7 | ↑ |
| A10 | CD 14 | 2843.8 | ↑ | 125.0 | ↑ | 11.8 | ↑ | 117.6 | ↑ |
| A11 | CD 30 | 731.7 | ↑ | 146.3 | ↑ | -26.1 | ↓ | 8.7 | ↑ |
| B2 | CD 40 Ligand | 940.8 | ↑ | -105.3 | ↓ | 44.4 | ↑ | 66.7 | ↑ |
| B3 | Chitinase 3-like 1 | 636.4 | ↑ | 325.2 | ↑ | 12.9 | ↑ | -57.6 | ↓ |
| B4 | CFD | 2357.1 | ↑ | -23.8 | ↓ | -114.3 | ↓ | -57.1 | ↓ |
| B5 | CRP | 1902.8 | ↑ | 69.4 | ↑ | -93.3 | ↓ | -66.7 | ↓ |
| B6 | Cripto-1 | 3500.0 | ↑ | 83.3 | ↑ | -66.7 | ↓ | -133.3 | ↓ |
| B7 | Cystatin C | 1555.6 | ↑ | 41.7 | ↑ | -200.0 | ↓ | -66.7 | ↓ |
| B8 | Dkk-1 | 2180.3 | ↑ | 139.3 | ↑ | -59.3 | ↓ | -81.5 | ↓ |
| B9 | DPPIV | 610.5 | ↑ | 232.6 | ↑ | -95.7 | ↓ | 69.6 | ↑ |
| B10 | EGF | 0.0 |  | -0.2 | ↓ | -0.5 | ↓ | -0.2 | ↓ |
| B11 | Emmprin | 50000.0 | ↑ | 12500.0 | ↑ | 35.3 | ↑ | 35.3 | ↑ |
| C2 | ENA-78 | 2309.5 | ↑ | -71.4 | ↓ | 571.4 | ↑ | 57.1 | ↑ |
| C3 | Endoglin | 2142.9 | ↑ | -142.9 | ↓ | -85.7 | ↓ | 28.6 | ↑ |
| C4 | Fas Ligand | 2875.0 | ↑ | 187.5 | ↑ | 0.0 |  | -200.0 | ↓ |
| C5 | FGF basic | 309.8 | ↑ | -18.1 | ↓ | -32.7 | ↓ | -93.9 | ↓ |
| C6 | FGF-7 | -1357.1 | ↓ | -571.4 | ↓ | -200.0 | ↓ | 0.0 |  |
| C7 | FGF-19 | 1696.1 | ↑ | 68.6 | ↑ | -76.9 | ↓ | -107.7 | ↓ |
| C8 | Flt-3 Ligand | -1666.7 | ↓ | -111.1 | ↓ | 800.0 | ↑ | -200.0 | ↓ |
| C9 | G-CSF | 4318.2 | ↑ | 363.6 | ↑ | 1200.0 | ↑ | 2400.0 | ↑ |
| C10 | GDF-15 | 31.0 | ↑ | 31.3 | ↑ | 15.8 | ↑ | -13.5 | ↓ |
| C11 | GM-CSF | -2642.9 | ↓ | -785.7 | ↓ | 80.0 | ↑ | 51.4 | ↑ |
| D1 | GROα | 210000.0 | ↑ | 52500.0 | ↑ | 58.1 | ↑ | -23.9 | ↓ |
| D2 | Growth Hormone | -1421.1 | ↓ | -52.6 | ↓ | -66.7 | ↓ | 0.0 |  |
| D3 | HGF | 4333.3 | ↑ | -333.3 | ↓ | -66.7 | ↓ | 200.0 | ↑ |
| D4 | ICAM-1 | 1209.7 | ↑ | -32.3 | ↓ | 61.5 | ↑ | -30.8 | ↓ |
| D5 | IFN-γ | 500.0 | ↑ | 5.7 | ↑ | -53.7 | ↓ | -39.0 | ↓ |
| D6 | IGFBP-2 | 14000.0 | ↑ | -500.0 |  | 200.0 | ↑ | 0.0 |  |
| D7 | IGFBP-3 | 20500.0 | ↑ | -500.0 | ↓ | 0.0 |  | -133.3 | ↓ |
| D8 | IL-1α | 831.9 | ↑ | 37.6 | ↑ | -28.6 | ↓ | -97.1 | ↓ |
| D9 | IL-1β | 2454.5 | ↑ | -227.3 | ↓ | 0.0 |  | -200.0 | ↓ |
| D10 | IL-1 ra | 2656.3 | ↑ | 62.5 | ↑ | -400.0 | ↓ | 400.0 | ↑ |
| D11 | IL-2 | 4666.7 | ↑ | -576.7 | ↓ | -200.0 | ↓ | 125.0 | ↑ |
| D12 | IL-3 | 3083.3 | ↑ | 1173.3 | ↑ | -200.0 | ↓ | 125.0 | ↑ |
| E1 | IL-4 | 1261.9 | ↑ | -71.4 | ↓ | -1000.0 | ↓ | 0.0 |  |
| E2 | IL-5 | 363.8 | ↑ | -46.7 | ↓ | 22.2 | ↑ | -88.9 | ↓ |
| E3 | IL-6 | 748.1 | ↑ | 26.7 | ↑ | 44.4 | ↑ | 71.1 | ↑ |
| E4 | IL-8 | 2684.2 | ↑ | 2671.1 | ↑ | 0.0 |  | -0.2 | ↓ |
| E5 | IL-10 | 1309.5 | ↑ | -47.6 | ↓ | -57.1 | ↓ | 171.4 | ↑ |
| E6 | IL-11 | 1253.5 | ↑ | 49.3 | ↑ | -200.0 | ↓ | 40.0 | ↑ |
| E7 | IL-12 p70 | 880.3 | ↑ | -28.2 | ↓ | -66.7 | ↓ | -133.3 | ↓ |
| E8 | IL-13 | 2083.3 | ↑ | -333.3 | ↓ | 133.3 | ↑ | 133.3 | ↑ |
| E9 | IL-15 | -10625.0 | ↓ | 0.0 |  | -133.3 | ↓ | -155.6 | ↓ |
| E10 | IL-16 | 2937.5 | ↑ | 125.0 | ↑ | -88.9 | ↓ | -44.4 | ↓ |
| E11 | IL-17A | 1478.0 | ↑ | 236.3 | ↑ | -59.5 | ↓ | 32.4 | ↑ |
| E12 | IL-18 Bpa | 22000.0 | ↑ | 2500.0 | ↑ | -200.0 | ↓ | -600.0 | ↓ |
| F1 | IL-19 | 1038.5 | ↑ | -76.9 | ↓ | -266.7 | ↓ | -200.0 | ↓ |
| F2 | IL-22 | 2093.8 | ↑ | 62.5 | ↑ | -160.0 | ↓ | 80.0 | ↑ |
| F3 | IL-23 | 4318.2 | ↑ | 227.3 | ↑ | -94.1 | ↓ | -58.8 | ↓ |
| F4 | IL-24 | 1805.6 | ↑ | 83.3 | ↑ | 146.7 | ↑ | 53.3 | ↑ |
| F5 | IL-27 | 746.9 | ↑ | -30.9 | ↓ | -141.2 | ↓ | -94.1 | ↓ |
| F6 | IL-31 | 4833.3 | ↑ | 166.7 | ↑ | 800.0 | ↑ | 0.0 |  |
| F7 | IL-32 | 3833.3 | ↑ | 23.8 | ↑ | -142.9 | ↓ | -114.3 | ↓ |
| F8 | IL-33 | 5083.3 | ↑ | -250.0 | ↓ | 600.0 | ↑ | -200.0 | ↓ |
| F9 | IL-34 | 4916.7 | ↑ | -333.3 | ↓ | -200.0 | ↓ | -266.7 | ↓ |
| F10 | IP-10 | -7125.0 | ↓ | -625.0 | ↓ | -200.0 | ↓ | -600.0 | ↓ |
| F11 | I-TAC | 1761.9 | ↑ | -47.6 | ↓ | -400.0 | ↓ | 400.0 | ↑ |
| F12 | Kallikrein-3 | 1074.5 | ↑ | 114.9 | ↑ | -30.3 | ↓ | -12.1 | ↓ |
| G1 | Leptin | 1531.3 | ↑ | -31.3 | ↓ | -40.0 | ↓ | -120.0 | ↓ |
| G2 | LIF | 3416.7 | ↑ | 83.3 | ↑ | -257.1 | ↓ | -114.3 | ↓ |
| G3 | Lipocalin-2 | 823.0 | ↑ | 373.9 | ↑ | -0.5 | ↓ | -22.4 | ↓ |
| G4 | MCP-1 | 1931.4 | ↑ | 186.3 | ↑ | -50.4 | ↓ | -60.7 | ↓ |
| G5 | MCP-3 | 3272.7 | ↑ | -45.5 | ↓ | 600.0 | ↑ | 0.0 |  |
| G6 | M-CSF | 3403.8 | ↑ | 76.9 | ↑ | 40.0 | ↑ | -240.0 | ↓ |
| G7 | MIF | 1500.0 | ↑ | 13.9 | ↑ | -32.7 | ↓ | -106.1 | ↓ |
| G8 | MIG | 3818.2 | ↑ | -45.5 | ↓ | 266.7 | ↑ | 66.7 | ↑ |
| G9 | MIP-1α/MIP-1β | -6375.0 | ↓ | -500.0 | ↓ | -266.7 | ↓ | 0.0 |  |
| G10 | MIP-3α | 91000.0 | ↑ | 14000.0 | ↑ | -400.0 | ↓ | 600.0 | ↑ |
| G11 | MIP-3β | -9125.0 | ↓ | -1125.0 | ↓ | -66.7 | ↓ | -66.7 | ↓ |
| G12 | MMP-9 | 1222.2 | ↑ | 152.8 | ↑ | -19.5 | ↓ | -46.6 | ↓ |
| H1 | Myeloperoxidase | 409.1 | ↑ | -20.7 | ↓ | 16.0 | ↑ | 0.0 |  |
| H2 | Osteopontin | 794.6 | ↑ | -35.7 | ↓ | 22.2 | ↑ | -22.2 | ↓ |
| H3 | PDGF-AA | 513.5 | ↑ | 4.5 | ↑ | -51.1 | ↓ | -51.1 | ↓ |
| H4 | PDGF-AB/BB | 1416.7 | ↑ | -250.0 | ↓ | -145.5 | ↓ | -109.1 | ↓ |
| H5 | Pentraxin-3 | 490.2 | ↑ | 46.3 | ↑ | -42.0 | ↓ | -44.4 | ↓ |
| H6 | PF-4 | 6000.0 | ↑ | -250.0 | ↓ | -400.0 | ↓ | -400.0 | ↓ |
| H7 | RAGE | 3062.5 | ↑ | -93.8 | ↓ | -200.0 | ↓ | 0.0 |  |
| H8 | RANTES | 1833.3 | ↑ | -47.6 | ↓ | -142.9 | ↓ | -57.1 | ↓ |
| H9 | RBP-4 | 1365.4 | ↑ | 57.7 | ↑ | 0.0 |  | -200.0 | ↓ |
| H10 | Relaxin-2 | 2263.9 | ↑ | 55.6 | ↑ | -600.0 | ↓ | 0.0 |  |
| H11 | Resistin | 641.8 | ↑ | 21.3 | ↑ | 44.4 | ↑ | 37.0 | ↑ |
| H12 | SDF-1α | 1467.4 | ↑ | 76.1 | ↑ | 155.6 | ↑ | -44.4 | ↓ |
| I1 | Serpin E1 | 543.0 | ↑ | 51.1 | ↑ | -0.7 | ↓ | -0.2 | ↓ |
| I2 | SHBG | 977.3 | ↑ | 0.0 |  | 114.3 | ↑ | -38.1 | ↓ |
| I3 | ST2 | 2071.4 | ↑ | 47.6 | ↑ | -40.0 | ↓ | -40.0 | ↓ |
| I4 | TARC | 4090.9 | ↑ | 272.7 | ↑ | 0.0 |  | 0.0 |  |
| I5 | TFF3 | 1166.7 | ↑ | -95.2 | ↓ | -142.9 | ↓ | 171.4 | ↑ |
| I6 | TfR | 1440.6 | ↑ | 84.2 | ↑ | -22.2 | ↓ | -44.4 | ↓ |
| I7 | TGF-α | 1531.3 | ↑ | -187.5 | ↓ | -114.3 | ↓ | -28.6 | ↓ |
| I8 | Thrombospondin-1 | 1031.3 | ↑ | 62.5 | ↑ | -15.1 | ↓ | -30.2 | ↓ |
| I9 | TNF-α | 1250.0 | ↑ | -57.7 | ↓ | -400.0 | ↓ | -400.0 | ↓ |
| I10 | uPAR | 2500.0 | ↑ | 166.7 | ↑ | -66.7 | ↓ | -200.0 | ↓ |
| I11 | VEGF | 2125.0 | ↑ | 31.3 | ↑ | 240.0 | ↑ | 40.0 | ↑ |
| J3 | Vitamin D BP | 1144.6 | ↑ | 93.4 | ↑ | -21.6 | ↓ | -43.2 | ↓ |
| J4 | CD-31 | 709.9 | ↑ | -6.2 | ↓ | -63.2 | ↓ | -94.7 | ↓ |
| J5 | TIM-3 | 1319.4 | ↑ | -13.9 | ↓ | -266.7 | ↓ | -66.7 | ↓ |
| J6 | VCAM-1 | 611.1 | ↑ | -24.7 | ↓ | -171.4 | ↓ | -85.7 | ↓ |
